# Proposal for targeted, neo-evolutionary-oriented, secondary prevention of early-onset endometriosis and adenomyosis. Part I: pathogenic aspects

**DOI:** 10.1093/humrep/dead229

**Published:** 2023-11-10

**Authors:** Paolo Vercellini, Veronica Bandini, Paola Viganò, Giorgia Di Stefano, Camilla Erminia Maria Merli, Edgardo Somigliana

**Affiliations:** Department of Clinical Sciences and Community Health, Academic Centre for Research on Adenomyosis and Endometriosis, Università degli Studi, Milano, Italy; Gynecology Unit, Fondazione IRCCS Ca’ Granda Ospedale Maggiore Policlinico, Milano, Italy; Department of Clinical Sciences and Community Health, Academic Centre for Research on Adenomyosis and Endometriosis, Università degli Studi, Milano, Italy; Department of Clinical Sciences and Community Health, Academic Centre for Research on Adenomyosis and Endometriosis, Università degli Studi, Milano, Italy; Gynecology Unit, Fondazione IRCCS Ca’ Granda Ospedale Maggiore Policlinico, Milano, Italy; Gynecology Unit, Fondazione IRCCS Ca’ Granda Ospedale Maggiore Policlinico, Milano, Italy; Gynecology Unit, Fondazione IRCCS Ca’ Granda Ospedale Maggiore Policlinico, Milano, Italy; Department of Clinical Sciences and Community Health, Academic Centre for Research on Adenomyosis and Endometriosis, Università degli Studi, Milano, Italy; Gynecology Unit, Fondazione IRCCS Ca’ Granda Ospedale Maggiore Policlinico, Milano, Italy

**Keywords:** endometriosis, adenomyosis, menstruation, ovulation, menarche, adolescence, ovarian cancer

## Abstract

The potential for repeated ovulation and menstruation is thought to have provided a Darwinian advantage during the Palaeolithic. Reproductive conditions remained relatively stable until the pre-industrial era, characterized by late menarche, very young age at first birth, multiple pregnancies, and prolonged periods of lactational amenorrhoea. For hundreds of thousands of years, menstruators experienced few ovulatory cycles, even though they were genetically adapted to ovulate and menstruate every month. In the post-industrial era, the age at menarche gradually declined, the age at first birth progressively increased, and breastfeeding became optional and often of short duration. This created a mismatch between genetic adaptation and socio-environmental evolution, so that what was initially a probable reproductive advantage subsequently contributed to increased susceptibility to diseases associated with lifetime oestrogen exposure, such as ovarian, endometrial and breast cancer and, hypothetically, also those associated with the number of ovulatory menstruations, such as endometriosis and adenomyosis. The incidence of endometriosis shows a steep and progressive increase around the age of 25 years, but given the consistently reported delay in diagnosis, the actual incidence curve should be shifted to the left, supporting the possibility that the disease has its roots in adolescence. This raises the question of whether, from an evolutionary point of view, anovulation and amenorrhoea should not still be considered the physiological state, especially in the postmenarchal period. However, an increase in the frequency of endometriosis in recent decades has not been demonstrated, although this deserves further epidemiological investigation. In addition, as endometriosis occurs in a minority of individuals exposed to retrograde menstruation, other important pathogenic factors should be scrutinised. Research should be resumed to explore in more detail the transtubal reflux of not only blood, but also endometrial cells, and whether they are systematically present in the peritoneal fluid after menstruation. If repetitive ovulatory menstruation during the early reproductive years is shown to increase the risk of endometriosis and adenomyosis development and progression in susceptible individuals, hormonal interventions could be used as secondary prevention in symptomatic adolescents.

## Introduction: connecting the dots

Endometriosis is defined as ‘*a disease characterized by the presence of endometrium-like epithelium and/or stroma outside the endometrium and myometrium, usually with an associated inflammatory process*’ (Becker *et al.*, 2022). According to the findings of systematic reviews, the prevalence of endometriosis is around 3–5% in the general female population of reproductive age, but around 30% in patients with infertility, 50% in patients with pelvic pain (Ghiasi *et al.*, 2020; Parazzini *et al.*, 2020; Sarria-Santamera *et al.*, 2020), and as high as 65–75% in symptomatic adolescents who do not respond to medical treatment (Janssen *et al.*, 2013; Hirsch *et al.*, 2020). The disease has a detrimental effect on health-related quality of life, psychological mood, sexual function, interpersonal and social relationships, working capacity, use of healthcare resources, and overall societal costs (Zondervan *et al.*, 2018, 2020; Bulun *et al.*, 2019; Taylor *et al.*, 2021; Horne and Missmer, 2022).

Adenomyosis is the presence of foci of endometrium within the myometrium, usually surrounded by hypertrophic smooth muscle cells and areas of fibrosis (Bulun *et al.*, 2021; Guo, 2023). Estimates of the prevalence of adenomyosis in menstruators of different ages vary widely, from <10% to >60%, depending on the presence of typical symptoms such as heavy menorrhagia and dysmenorrhoea, physician awareness of the condition, the accuracy of the diagnostic techniques adopted, and the interpretation of imaging findings (Vercellini *et al.*, 2006; Upson and Missmer, 2020).

Endometriosis and adenomyosis often co-exist (Bulun *et al.*, 2023), can be diagnosed clinically (i.e. non-surgically) with the support of transvaginal ultrasonography or, in selected circumstances, pelvic MRI (Vercellini *et al.*, 2006; Upson and Missmer, 2020), and are associated with adverse obstetric outcomes (Vercellini *et al.*, 2023).

Various theories have been proposed to explain the development of endometriosis, but the metastatic model, based on the implantation of endometrial cells reaching the pelvis via transtubal retrograde flow during menstruation, is supported by the largest body of evidence (Vercellini *et al.*, 1998, 2007, 2014; Zondervan *et al.*, 2018, 2020; Bulun *et al.*, 2019; Taylor *et al.*, 2021; Horne and Missmer, 2022). Different pathogenic hypotheses have also been suggested for adenomyosis, but awareness and interest in the condition have only recently increased and diagnostic capabilities are rapidly improving. This has led to a change in the epidemiological scenario and adenomyosis is now considered to be a disease not only of parous menstruators in their forties, but also of young menstruators (Martire *et al.*, 2020; Bulun *et al.*, 2021; Guo 2023; Millischer *et al.*, 2023).

Although additional factors must play a role in the progression of the two diseases, a high number of consecutive ovulatory menses could theoretically contribute to the early phase of development of both conditions by facilitating (i) endometrial pelvic contamination via transtubal retrograde blood flow and (ii) endometrial invasion of the inner myometrium via recurrent trauma and inflammation at the endo-myometrial junction (Bulun *et al.*, 2019, 2021, 2023; Guo, 2020; Maruyama *et al.*, 2020; Bulun, 2022). According to some investigators, adenomyosis and endometriosis may be different phenotypes of a single disorder resulting from inappropriate and dysfunctional myometrial contractions during menstruation caused by a chronic intrauterine inflammatory state (Leyendecker *et al.*, 1998, 2009, 2015, 2023; Maruyama *et al.*, 2020).

Several years ago, our group hypothesized a common pathogenesis for sporadic serous, endometrioid, and clear cell ovarian cancer, proposing as a shared carcinogenic pathway the oxidative stress induced by iron overload resulting from reiterative retrograde menstruation (Vercellini *et al.*, 2011). Our model, initially based mainly on epidemiological evidence, was subsequently shown to be compatible with laboratory findings reported by independent investigators (Seidman, 2013; Lattuada *et al.*, 2015; Huang *et al.*, 2016; Uberti *et al.*, 2016; Rockfield *et al.*, 2019; Chhabra *et al.*, 2021). Indeed, the presence of blood in the peritoneal fluid has been recognized as a likely oncogenic factor (Lin *et al.*, 2017; Rockfield *et al.*, 2017; Dawson *et al.*, 2018; Otsuka, 2021).

We now revisit our hypothesis with the aim of proposing a unifying theory that includes endometriosis and adenomyosis in addition to the above ovarian cancer histotypes. The underlying concept remains the notion of repetitive ovulatory menstruation (ROM) as a source of reiterative bleeding and thus as a direct or indirect spring of iron-induced oxidative stress and secondary chronic inflammation (Van Langendonckt *et al.*, 2002a,b; Lousse *et al.*, 2009, 2012; Donnez *et al.*, 2016; Bulun *et al.*, 2019, 2021; Wyatt *et al.*, 2023). However, whereas the ovarian cancer model takes into account the total number of ovulatory menstrual cycles over the entire reproductive period (Eaton *et al.*, 1994, 2002; Fu *et al.*, 2022), and thus the lifetime pelvic exposure to blood, in the case of endometriosis and adenomyosis the focus is on early postmenarchal exposure (Kvaskoff *et al.*, 2013; Martire *et al.*, 2020; Bulun *et al.*, 2021; Millischer *et al.*, 2023). Epidemiological and endocrinological factors will be interpreted here in the light of recent changes in menstrual and reproductive patterns (Short, 1976, 1994; Thomas and Ellertson, 2000; Renfree, 2012; Fathalla, 2019).

In the first part of this opinion piece, we hypothesize that endometriosis and adenomyosis may partly result from a mismatch between Darwinian genetic adaptation and global social evolution (Thomas, 1993; Eaton *et al.*, 1994, 2002; Fathalla, 2019; Pei *et al.*, 2022). As a consequence of an unprecedented increase in the number of ovulatory menstruations in the postmenarchal years, iron overload caused by repetitive bleeding episodes would act as a major trigger for chronic inflammation as well as tissue injury and repair by fibrosis, thus providing a common thread for the aetiology, pathogenesis, clinical manifestations, and potential complications of early-onset endometriosis and adenomyosis (Lousse *et al.*, 2012; Donnez *et al.*, 2016; Bulun *et al.*, 2019, 2021, 2023; Guo 2020, 2023; Maruyama *et al.*, 2020; Bulun, 2022; Kobayashi *et al.*, 2023; Wyatt *et al.*, 2023).

Information was identified by searching PubMed using the MESH terms ‘endometriosis’ and ‘adenomyosis’ in combination with ‘menstruation’, ‘epidemiology’, ‘etiology’, ‘pathogenesis’, ‘infertility’, ‘pain’, ‘oral contraceptives’, ‘progestins’, and ‘ovarian cancer’. References from relevant publications were systematically screened and further articles identified using PubMed’s ‘similar articles’ and ‘cited by’ functions. The search was limited to peer-reviewed, full-text, and English language articles. For this opinion article, priority was given to systematic reviews and meta-analyses, as well as original studies published in journals currently rated as Q1 (i.e. first quartile or the top 25%) by ISI Web of Science/Clarivate in the category ‘Clinical Medicine’ and the subcategory ‘Obstetrics & Gynaecology’. Specific international guidelines were also consulted.

A detailed critical review of the many pathogenic mechanisms that may underlie the development and progression of endometriosis and adenomyosis is beyond the scope of this article, and readers are referred to the several excellent publications available on the subject (Zondervan *et al.*, 2018, 2020; Bulun *et al.*, 2019, 2021, 2023; Guo 2020, 2023; WaNg *et al.*, 2020; Taylor *et al.*, 2021; Horne and Missmer, 2022).

## What does social evolution have to do with endometriosis and adenomyosis?

From a health care perspective, the transition from the pre-industrial to the post-industrial era has been characterised by a shift from a predominantly acute disease pattern (e.g. infections) to a predominantly chronic disease pattern (i.e. degenerative diseases) (Eaton *et al.*, 2002). Many anthropologists, evolutionary biologists, and geneticists support the concept that much of this is due to a mismatch between extremely slow Darwinian genetic adaptation on the one hand, and recent extremely rapid environmental and social evolution on the other (Short, 1976; Strassmann, 1996, 1997; Eaton *et al.*, 1994, 2002; Thomas and Ellertson, 2000; Renfree, 2012; Fathalla, 2019; Pei *et al.*, 2022). In short, it took millions of years for humans to undergo selective genetic adaptation that favoured specific traits that were advantageous in those environmental and social conditions. However, when living conditions changed rapidly, the same genetic characteristics became disadvantageous and contributed to the spread of previously uncommon diseases (Eaton *et al.*, 1994, 2002).

The potential for repeated ovulation and menstruation probably provided a Darwinian advantage during the Palaeolithic (Jarrell, 2018), although the real reason why people began to menstruate regularly is still debated (Finn, 1996). In fact, only humans and a few other mammals (e.g. apes, Old World monkeys, and shrews) menstruate (Critchley *et al.*, 2020a; Strassmann, 1996). Reproductive conditions (i.e. the environment) remained relatively stable until the pre-industrial times, and were characterized by late menarche, very young age at first birth, multiple pregnancies, and prolonged periods of lactational amenorrhoea (Short, 1976, 1994; Eaton *et al.*, 1994, 2002; Strassmann, 1996, 1997). As a result, for hundreds of thousands of years, menstruators experienced few ovulatory menses, even though they were genetically adapted to potentially ovulate and menstruate every month. In a traditional West African population in Mali, regular menstruation is still a sign of sterility, not fertility (Strassman, 1999).

In the post-industrial era, living conditions improved greatly and the role of menstruators in modern societies evolved rapidly. Thus, in less than two centuries, the age at menarche gradually declined, the age at first birth progressively increased, and breastfeeding became optional and often of short duration (Haines and Guest, 2008; Table 1). This created a mismatch between genetic adaptation and socio-environmental evolution, so that what was initially a reproductive advantage (Finn, 1996; Jarrell, 2018) subsequently contributed to increased susceptibility to diseases associated with lifetime oestrogen exposure, such as ovarian, endometrial, and breast cancer (Jordan *et al.*, 2012; Schüler *et al.*, 2013; Bieuville *et al.*, 2023).

**Table 1.** Variation in reproductive pattern and estimated number of ovulatory menstruations in different periods of reproductive life during the last two centuries in Western countries. Literature data.*

Variable	Nineteenth century	Twenty-first century
Menarche	16 years	12.5 years
Mean no. of children per woman	5–6	1–2
Mean duration of exclusive breast lactation	1–2 years	4–6 months
Mean no. of menstruations between menarche and 25 years of age	∼50	∼150
Mean no. of menstruations during the menarche-to-FFTP interval	∼20	∼200
Mean no. of lifetime ovulatory menstruations	40–160	400–460

* Data from Short (1976, 1994), Wyshak and Frisch (1982), Eaton (1994, 2002), Thomas (1993), Strassmann (1996, 1997), Haines and Guest (2008), Fathalla (2019), InterLACE Study Team (2019), and Gottschalk *et al.* (2020).

FFTP, first full-term pregnancy.

In this context, repetitive ovulation and menstruation without prolonged periods of amenorrhoea induced by multiple pregnancies and prolonged lactation may be considered a maladaptive reproductive trait that may also increase the risk of diseases such as endometriosis and adenomyosis (Renfree, 2012; Fathalla, 2019; Pei *et al.*, 2022). In other words, it cannot be excluded that the exposure to repeated menstruation-related and blood-mediated tissue injury and repair and monthly transtubal mentrual reflux may be greater than the female immune system has genetically evolved to handle at the endo-myometrial junction or within the pelvic environment, respectively (Guo, 2020; Maruyama *et al.*, 2020; Kobayashi, 2023).

### Is monthly menstruation for years on end the physiological norm?

In 1976, Roger Short reviewed the available evidence on the evolution of human reproduction and the secular changes in environmental and nutritional conditions, sexual behaviours, social organization, and biological events that regulate age at menarche, conception, and lactation, i.e. the factors that control the interval between successive births and the lifetime menstrual pattern over subsequent periods of human history (Short, 1976). Based on anthropological and evolutionary data, the author reported that in hunter-gatherer communities a girl became pregnant ∼3 years after puberty. Lactational amenorrhoea lasted ∼3 years, and another pregnancy usually occurred after a few ovulatory cycles. Because of limited life expectancy, a menstruator generally gave birth to an average of five children and spent most of their reproductive life without menstruation (Short, 1976, 1994). Nowadays, individuals from Western, industrialized, high-income nations give birth to an average of two children or less, with limited breastfeeding, and life expectancy has largely overcome the menopause insurgency. As a result, 35 years of their reproductive life are characterized by regular menstrual cycles. Short (1976) concluded: ‘*There can be no doubt that this ninefold increase in the time spent having menstrual cycles poses a number of new problems for us; it is something of which we have had no prior evolutionary experience, and hence we are not genetically adapted to cope with the situation*’. Italy is a case in point: the average age at first birth rose by 7.5 years between 1974 and 2022, and the average number of children per menstruator gradually fell from 2.7 in 1964 to 1.2 in 2022. The average duration of exclusive breastfeeding is now ∼4 months (Istituto Nazionale di Statistica, 2023). However, in non-Western, industrialized, high-income societies, it is still common for more than two children to be the norm.

The evolutionary view of Short (1976, 1994), has lately been shared by several other researchers (Eaton *et al.*, 1994, 2002; Strassmann, 1996, 1997; Thomas and Ellertson, 2000; Renfree, 2012; Fathalla, 2019), including Pei *et al.* (2022), who also reviewed more recent evidence on the potential effects of ROM and excess oestrogen exposure. However, while the associations between ROM and risk of endometrial, ovarian, and breast cancer are strong and support causation (Jordan *et al.*, 2012; Schüler *et al.*, 2013; Fu *et al*, 2022; Bieuville *et al.*, 2023), the relationship with endometriosis is less clear.

An inverse association between higher parity and the risk of endometriosis has been consistently observed (Missmer and Cramer, 2003; Viganò *et al.*, 2004; Parazzini *et al.*, 2017; Shafrir *et al.*, 2018). In a case–control study conducted in Lombardy, northern Italy (Parazzini *et al.*, 1995), the punctual odds ratio (OR) of endometriosis compared with nulliparous menstruators was 0.4 in menstruators reporting 1 birth and 0.2 in those reporting ≥2 births, thus confirming the results of another case–control study previously conducted in the same region (Candiani *et al.*, 1991). Analysing data from 473 patients who underwent surgery for various indications, Peterson *et al.* (2013) observed a reduced risk of endometriosis in parous compared to nulliparous individuals (adjusted OR 0.27; 95% CI 0.15–0.49). The risk was even lower when the analysis was restricted to patients with moderate or severe endometriosis only (adjusted OR 0.19; 95% CI 0.10–0.37).

The effect of reproductive events on the risk of endometriosis was investigated by Missmer *et al.* (2004) using prospective data from the Nurses’ Health Study II cohort. A higher incidence of laparoscopically diagnosed endometriosis was observed in individuals with an earlier age at menarche and shorter cycle length during adolescence. A linear decrease in risk was also observed with the number of live births and lifetime breastfeeding duration. There was also a significant association between the number of lifetime ovulatory cycles and the risk of endometriosis. Importantly, among people who had never used combined oral contraceptives, the risk of endometriosis was 6-fold in the highest quartile of lifetime cycles (>291) compared with people in the lowest quartile (<174) (Missmer *et al.*, 2004).

The inverse association between duration of total and exclusive breastfeeding and incident endometriosis was further confirmed by Farland *et al.* (2017a), who observed an 8% reduction in endometriosis risk for each additional 3 months of total breastfeeding and a 14% lower risk for each additional 3 months of exclusive breastfeeding. Compared with individuals who never breastfed, those who breastfed for more than 3 years had a 40% reduced risk of endometriosis. However, the association was only partly influenced by postpartum amenorrhoea. This period is characterized by several metabolic phenomena that may play a role, including a trend towards postpartum weight loss, a recognized maternal health benefit of breastfeeding (Hart *et al.*, 2022).

Regarding menstrual characteristics, Cramer *et al.* (1986) first reported that surgical patients with short cycle length (≤27 days) and long flow (≥7 days) had more than double the risk of endometriosis compared to patients with longer cycle length and shorter flow. Also in the study by Darrow *et al.* (1993), when the analysis was restricted to subjects <30 years of age and using age-matched friend controls, menstruators with flow ≥6 days per month and those with heavy flows had more than a 2-fold increased risk of endometriosis (OR 2.5; 95% CI 1.1–5.9 and OR 2.5; 95% CI 1.1–6.3, respectively). Parazzini *et al.* (1995) observed that, compared with patients who reported lifelong regular menstrual cycles, the OR in those with irregular cycles was 0.4 (95% CI 0.2–0.8). In the case–control study by Sangi-Haghpeykar and Poindexter (1995), a long duration of uninterrupted menstrual cycles was a risk factor for endometriosis (OR 2.9; 95% CI 1.3–6.4). Similar results were observed in the Yale series, where patients with endometriosis reported significantly shorter cycles and longer and heavier flow than controls (Matalliotakis *et al.*, 2008).

Overall, the available evidence on reproductive and menstrual patterns supports the notion that augmented exposure to ROM increases the risk of endometriosis. This is compatible with increased pelvic contamination by refluxed endometrium (Shafrir *et al.*, 2018; Missmer and Cramer, 2003). The effect of pregnancy may also be explained by the protective action of high progesterone levels in addition to the prolonged period of amenorrhoea (Parazzini *et al.*, 2017). In the pre-industrial era, multiple pregnancies meant the creation of a progesterone-dominant environment for much of the decade following menarche (Short, 1976, 1994; Renfree, 2012). The effect of progesterone may be particularly important in the early phase of endometriosis development (WaNg *et al.*, 2020; Shafrir *et al.*, 2023), as it may inhibit the sequence of events leading to lesion development and progression.

### Are endometriosis and adenomyosis rooted in adolescence?

**Figure 1.**
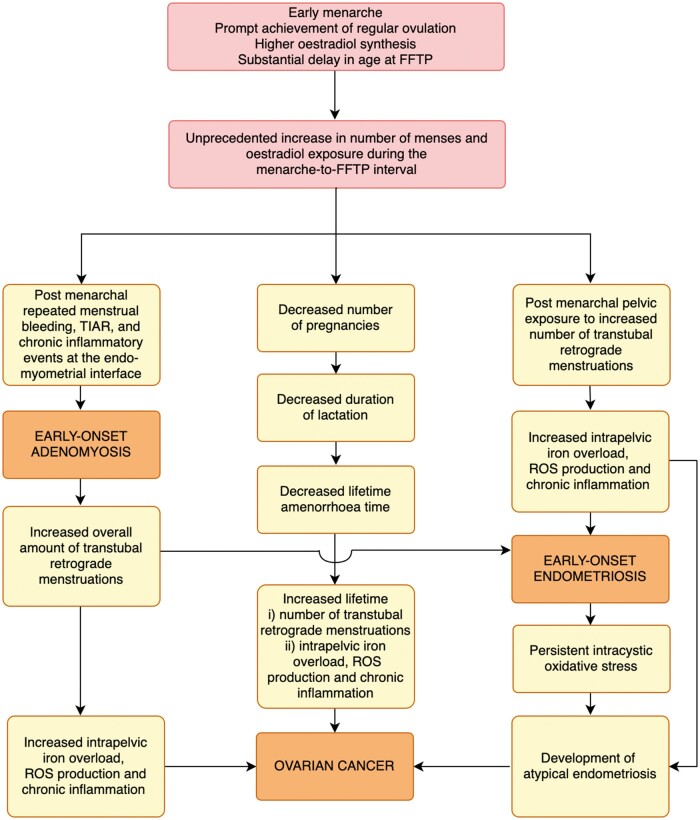
**The repetitive menstruation hypothesis revisited.** A neo-evolutionary theoretical framework based on the effect of a common pathogenic pathway on the risk of endometriosis, adenomyosis and ovarian cancer. FFTP, first full-term pregnancy; TIAR, tissue injury and repair; ROS, reactive oxygen species.

The potential impact of the substantial increase in the number of ovulatory menstrual cycles over the past two centuries should be considered separately for oncological and benign diseases (Fig. 1). The risk of ovarian, endometrial, and breast cancer, the peak incidence of which occurs after menopause, is influenced by the cumulative number of ovulations and menstruations over a lifetime, which is also a reasonable proxy for lifetime oestrogen exposure (Eaton *et al.*, 1994, 2002; Jordan *et al.*, 2012; Schüler *et al.*, 2013; Bieuville *et al.*, 2023).

However, the peak incidence of oestrogen-dependent benign diseases such as endometriosis and adenomyosis occurs much earlier (Missmer and Cramer, 2003; Shafrir *et al.*, 2018; Parazzini *et al.*, 2020; Upson and Missmer, 2020). In particular, after a steep and progressive increase around the age of 25 years, the incidence curve of endometriosis tends to plateau after 5 years (Parazzini *et al.*, 2017, 2020). Such a curve represents cases when they are diagnosed, not when they actually develop. Thus, given the consistently reported delay in diagnosis (Davenport *et al.*, 2023), the actual incidence curve should realistically be shifted to the left (Kvaskoff *et al.*, 2013). Therefore, the period of highest risk appears to be from menarche to the age of 25 years, i.e. the years preceding the detection of the majority of full-blown endometriosis cases (Viganò *et al.*, 2004; Kvaskoff *et al.*, 2013; Parazzini *et al.*, 2017, 2020; Shafrir *et al.*, 2018; Koninckx *et al.*, 2021b).

Based on available data on the prevalence of endometriosis in the general population, menstruators could be divided into susceptible (∼5%) and non-susceptible (∼95%) subgroups (Parazzini *et al.*, 2017, 2020; Koninckx *et al.*, 2021b). In light of the above observations, susceptible individuals may develop endometriosis after a limited period of pelvic exposure to retrograde menstruation (Kvaskoff *et al.*, 2013; Koninckx *et al.*, 2021b). Thus, over time, predisposed persons would be selectively excluded from the average-risk general female population, which would become increasingly composed of non-susceptible individuals. This would explain the non-linearity of the incidence curve and its plateau after the age of 30 years (Parazzini *et al.*, 2017, 2020; Koninckx *et al.*, 2021b).

In the same vein, Koninckx *et al.* (2021b) suggests that the growth period of endometriotic lesions is rather short. In a series of 2086 patients who underwent laparoscopy for endometriosis at the University Hospital Gasthuisberg between 1988 and 2011, the number of surgical patients increased up to the age of 28 years and decreased thereafter. In addition, the severity and relative frequency of different endometriotic lesions did not vary in patients aged 24–44 years.

If endometriosis has its roots in adolescence, efforts to prevent the onset or progression of the disease should focus on the first few postmenarchal years (Kvaskoff *et al.*, 2013). In Western countries, the average age at menarche decreased from 15 to 17 years for menstruators born in the early 1800s to 12.6 years for those born between 1970 and 1984 (Wyshak and Frisch, 1982; InterLACE Study Team, 2019; Gottschalk *et al.*, 2020). As nowadays the average age at first birth is well over 25 years in most nations (United Nations Department of Economic and Social Affairs, Population Division, 2022), the number of ovulatory menstruations during the interval between menarche and peak endometriosis incidence in individuals not using hormonal contraception has approximately tripled (Table 1). Indeed, in European countries, the time from menarche to first full-term pregnancy (menarche-to-FFTP interval) has increased in two centuries from only 3 years (∼16 years at menarche and ∼19 years at first birth; Short, 1976, 1994; Eaton *et al.*, 2002) to almost 20 years. In Italy, for example, the age at menarche is 12.4 years (De Sanctis *et al.*, 2019; Piras *et al.*, 2020) and the age at first birth is 32.4 years (Istituto Nazionale di Statistica, 2023).

Moreover, given the inverse relationship between decreasing age at menarche and the number of ovulatory menstrual cycles, as well as the amount of gonadal oestradiol (E2) synthesis in the postmenarchal period (Macmahon *et al.*, 1982; Vihko and Apter, 1984; Farland *et al.*, 2017b), such an impressive increase in the length of the menarche-to-FFTP interval may have an even greater effect on the risk of developing endometriosis than simply augmenting the number of menstruations. In fact, compared with late menarche, early menarche is associated with higher serum FSH and E2 concentrations and more frequent ovulatory cycles (Vihko and Apter, 1984). Thus, not only are individuals with early menarche exposed to oestrogen for a longer period of time, but this excess exposure is characterised by particularly high serum oestrogen levels (Macmahon *et al.*, 1982; Emaus *et al.*, 2008; Farland *et al.*, 2017b).

According to Lund *et al.* (2022), early menarche can be seen as a proxy measure for increased oestrogen exposure during adolescence. Even in the case of partially anovulatory cycles, proliferation of refluxed endometrial cells would be favoured by unopposed exposure to E2 action that is not counterbalanced by progesterone action (Gunn *et al.*, 2018; Carlson and Shaw, 2019). In addition to local stimulation of eutopic and ectopic endometrium, oestrogens also exert a systemic pro-inflammatory effect (Straub, 2007; Oertelt-Prigione, 2012a,b; Cutolo *et al.*, 2014; Dodd and Menon, 2022).

Along this line, a recent meta-analysis of data published over the last two decades showed that menarche before the age of 12 years was associated with a one-third increased risk of endometriosis (OR 1.34; 95% CI 1.16–1.54) (Lu *et al.*, 2023). When only studies started after 2000 were considered, the risk increased further. The risk was particularly high in low-resource countries (OR 2.11; 95% CI 1.55–2.87). These results confirm the findings of a previous systematic review and meta-analysis showing that, based on selected studies with rigorous control of confounders, early age at menarche was significantly associated with a higher risk of endometriosis (Nnoaham *et al.*, 2012). In the case–control study by Treloar *et al.* (2010), menarche after the age of 14 years of age was strongly and inversely associated with endometriosis (OR 0.3; 95% CI 0.1–0.6).

African-American girls experience menarche at an earlier age than Caucasians. In the study conducted by Reagan *et al.* (2012) on a sample of 23 337 girls drawn from the US National Longitudinal Survey of Youth Child–Mother file, the mean age at menarche was 144 months for African-American adolescents and 150 months for whites. This would disproportionately expose the former subpopulation to the possible deleterious effects of early ROM, potentially exacerbating the racial and ethnic health disparities in endometriosis diagnosis and management already observed in the USA (Bougie *et al.*, 2019, 2022; Orlando *et al.*, 2022; Westwood *et al.*, 2023). In this context, given the difficulties in diagnosis, the apparent lack of differences in the incidence of endometriosis between ethnic groups (Katon *et al.*, 2023) may actually reflect differences in access to care (Williams *et al.*, 2019; Westwood *et al.*, 2023).

### A word of caution

There are three ways to interpret the presented epidemiological evidence: (i) there are direct biological links between early menarche, late age at first birth, low total number of live births, and shortened breastfeeding to endometriosis and adenomyosis, independently; (ii) each or some of these factors are proxies for higher ROM, which is the causal biological link to endometriosis and adenomyosis; or (iii) most likely, there is a combination of (i) and (ii). In the following sections, we will present biological and molecular arguments as to why these epidemiological factors may be proxies for ROM, which in turn acts as the triggering mechanistic event for the onset and progression of endometriosis and adenomyosis.

It should be noted, however, that the social evolution referred to here is specific to post-industrialized, generally high-income, nations. It is therefore critical not to overgeneralize this theory to the whole world, but to limit it to the specific circumstances (i.e. societies) in which it is relevant. In this regard, Henrich *et al.* (2010) coined the acronym WEIRD (i.e. Western, Educated, Industrialised, Rich, and Democratic) with the aim of raising awareness of humanity’s cultural diversity and encouraging scientists to differentiate their sampling. Recognizing that WEIRD populations are a limited subset of the total world population could avoid sampling bias and inappropriate generalisations.

In addition, despite the above findings, it may still be difficult to disentangle the evolutionary issues from other epidemiological aspects (Eaton *et al.*, 2002), and since retrograde menstruation is reported to be a common phenomenon in most menstruators (Halme *et al.*, 1984; Liu and Hitchcock, 1986; Tang *et al.*, 2022), multiple factors in addition to ROM are likely to play a role in the development of endometriosis and adenomyosis (Shafrir *et al.*, 2018; Zondervan *et al.*, 2018, 2020; Koninckx *et al.*, 2019; Leonardi *et al*, 2020; Olšarová and Mishra, 2020; WaNg *et al.*, 2020; Kuan *et al.*, 2021; Salliss et al., 2021; Taylor *et al.*, 2021; Horne and Missmer, 2022; Marroquin *et al.*, 2023; Muraoka *et al.*, 2023; Vallée *et al.*, 2023; Yang *et al.*, 2023). Even an increase in endocrine-disrupting chemicals due to air and water pollution and occupational hazards can potentially lead to the onset and progression of endometriosis (Vallée *et al.*, 2023). Exposure to these contributors, including environmental, sociological, and microbiological risk factors, may change over time or vary between geographical regions, potentially influencing the development of endometriosis (Ghiasi *et al.*, 2020). Furthermore, several morbidities are associated with early menarche, some of which may be explained by more menstrual cycles, but also by other conditions that would cause early menarche, such as elevated leptin levels, particularly in adolescents with a metabolically unhealthy phenotype (Gamble, 2017).

## Ovulation and menstruation as inflammatory events

The hypothesis of an effect of ROM on the risk of endometriosis and adenomyosis maybe even more plausible if one considers not only the number of events, in terms of the mere total amount of displaced endometrial tissue and thus its probabilistic chance of survival at ectopic sites, but also the quality of such events, in terms of a possible transition from a state of acute and self-limited inflammation to a state of chronic and self-perpetuating inflammation (Guo, 2018, 2020, 2023; Bulun *et al.*, 2019, 2021; Kobayashi, 2023; Kobayashi *et al.*, 2023). In this context, both ovulation and menstruation can be considered as a source of acute inflammation with a risk of chronicity if relentlessy repeated (Jarrell and Arendt-Nielsen, 2016; Jarrell, 2018).

An LH-triggered inflammatory process is the mechanism by which the connective tissue layers of the tunica albuginea and theca externa at the apex of a pre-ovulatory follicle are loosened, allowing the follicle wall to migrate towards the ovarian surface and then rupture due to intrafollicular fluid pressure (Espey, 1994; Richards *et al.*, 2002; Gérard *et al.*, 2004). The fundamental role of inflammation during ovulation is supported by the observation that pre-ovulatory use of non-steroidal anti-inflammatory drugs and cyclo-oxygenase (COX)-2 inhibitors can interfere with this process to the point of reducing the likelihood of conception (Weiss and Ghandi, 2016; Duffy *et al.*, 2019). Li *et al.* (2023) recently demonstrated that piroxicam, a COX-2 inhibitor, confers a significant synergistic contraceptive effect to levonorgestrel used for emergency contraception.

The potential impact of repeated, ovulation-associated local inflammation on ectopic endometrial pelvic implants is currently unknown. However, with each ovulation, large amounts of intrafollicular fluid E2 are released directly onto pelvic lesions, potentially promoting disease progression (Bulun *et al.*, 2019; Koninckx *et al.*, 2021c; Koninckx *et al.*, 2022). The role of ovulation in the pathogenesis of ovarian endometriomas is indirectly confirmed by the remarkable protective effect of prolonged postoperative hormonal therapy on endometrioma recurrence rates. In the systematic review and network meta-analysis by Chiu *et al.* (2022), the OR of cyst recurrence in patients who underwent ovulation suppression compared with those who chose expectant management varied from 0.04 to 0.21, depending on the type of therapy used.

The relationship between lifetime ovulatory years and risk of epithelial ovarian cancer has also been interpreted in terms of reiterative inflammatory events (Ness and Cottreau, 1999). This may be particularly relevant for patients with endometriosis as, according to the results of a meta-analysis of 24 cohort and case–control studies in which endometriosis-related tumours where overrepresented (Kvaskoff *et al.*, 2021), the absolute lifetime risk of developing ovarian cancer was 2.5% (1 in 40 individuals) in patients with endometriosis, compared with 1.3% (1 in 75 individuals) observed in the general population (Reid *et al.*, 2017). Indeed, based on the findings of another systematic review and meta-analysis (Fu *et al.*, 2022), the strength of the association between lifetime ovulations and ovarian cancer was higher for endometrioid tumours (pooled OR 3.05; 95% CI 2.08–4.45) than for serous tumours (pooled OR 2.31, 95% CI 1.60–3.33). According to the dualistic model of epithelial carcinogenesis, endometrioid tumours of the ovary are included in the group of tumours arising from endometriosis (Kurman and Shih, 2016).

Menstruation should also be considered an inflammatory phenomenon (Jarrell, 2018), with histological and biological features similar to those classically observed when inflammation occurs at any other site in the body (Finn, 1986; Critchley *et al.*, 2020b; Jain *et al.*, 2022). The decline in ovarian steroid concentration prior to the onset of menses leads to increased synthesis of ROS, prostaglandins, cytokines, chemochines, and matrix metalloproteinases (Evans and Salamonsen, 2012), as well as interleukin (IL)-1β and IL-18, which can also lead to systemic inflammatory events (Azlan *et al.*, 2020).

Under physiological conditions, menstruation-associated inflammation is self-limited and does not lead to persistent inflammation with the consequences of tissue destruction, fibrotic replacement, and potential loss of function (Critchley *et al.*, 2020b). However, some researchers have suggested that ROM may sometimes induce chronic inflammation, uterine hypercontractility, and fibrotic scarring due to repeated myometrial injury and repair (Bulun *et al.*, 2021; Guo, 2023; Kobayashi, 2023).

Thus, if ovulation and menstruation are acute inflammatory events, their endless repetition may not necessarily be considered physiological, and the question may arise as to whether their incessant occurrence in the early reproductive period may favour the onset of endometriosis and adenomyosis, and through what pathogenic pathways (Bulun *et al.*, 2019, 2021, 2023).

## Does ROM contribute to the early-onset and progression of endometriosis and adenomyosis?

The group of Jacques Donnez carried out pioneering studies on dysregulated iron homeostasis in the pelvic cavity, which would promote oxidative stress and inflammation (Van Langendonckt *et al.*, 2002a,b; Lousse *et al.*, 2009). Erythrocytes refluxed into the pelvis during menstruation are normally phagocytized and lysed by local macrophages (Lousse *et al.*, 2012; Donnez *et al.*, 2016). However, in an oversimplified synthesis, when the capacity of pelvic macrophages to eliminate erythrocytes and haemoglobin is overwhelmed, or the homeostatic processes are defective, toxic by-products such as haem and free iron are released (Lousse *et al.*, 2012; Donnez *et al.*, 2016; Kobayashi *et al.*, 2023; Wyatt *et al.*, 2023). The resulting Fenton reaction and generation of ROS promotes an inflammatory state, with damage to the peritoneal mesothelium, exposure of the underlying mesenchymal tissue, and the possibility of implantation of regurgitated endometrial cells (Lousse *et al.*, 2009, 2012; Donnez *et al.*, 2016; Ng *et al.*, 2020; Ansariniya *et al.*, 2022). This may be particularly likely to occur in menstruators with increased pelvic exposure to blood and regurgitated endometrial glands, such as those with regular cycles and abundant menstrual flow (Darrow *et al.*, 1993; Vercellini *et al.*, 1997; Missmer and Cramer, 2003; Parazzini *et al.*, 2017), or in those with inadequate pelvic scavenging systems (e.g. haptoglobin, which binds extracellular haemoglobin and facilitates its clearance by macrophages, and haem oxygenase, which degrades haemoglobin and haem with incorporation of iron into ferritin in pelvic macrophages) (Van Langendonckt *et al.*, 2002a; Lousse *et al.*, 2009, 2012; Pirdel and Pirdel, 2014; Donnez *et al.*, 2016; Wyatt *et al.*, 2023). Indeed, the presence of haemosiderin-laden macrophages (siderophages) is a histological hallmark of endometriosis and supports the hypothesis that peritoneal antioxidant mechanisms may have been overwhelmed (Lousse *et al.*, 2009, 2012; Donnez *et al.*, 2016). The recent systematic review by Wyatt *et al.* (2023) summarizes the available evidence on the role of abnormal pelvic iron homeostasis due to retrograde menstruation in the development and progression of endometriosis.

Furthermore, oestrogens are a biological driver of inflammation in endometriosis, as E2 is crucial for endometrial gland survival, with subsequent production of a number of inflammatory molecules leading to peritoneal and subperitoneal tissue remodelling, adhesions, and fibrosis (Bulun *et al.*, 2012, 2019; Reis *et al.*, 2013; Clemenza *et al.*, 2022). In this regard, using data from the Nurses’ Health Study II, Shafrir *et al.* (2023) found that menstruators with higher early follicular phase free or total plasma E2 levels had an increased risk of surgically confirmed endometriosis at least 1 year after blood sampling. In addition, higher mid-luteal plasma progesterone levels were associated with a lower risk of endometriosis.

These findings support the notion that repeated retrograde menstruation may act as an initiating factor in the pathogenesis of endometriosis (Jarrell, 2018), and that promoting factors, including dysfunctional pelvic iron homeostasis, high oestrogen exposure, and reduced progesterone exposure, may determine disease development and progression in genetically susceptible individuals (Parazzini *et al.*, 2017).

Repetitious menstrual episodes also imply reiterative intrauterine tissue hypoxia and necrosis, myometrial contractions, and rapid angiogenesis and regenerative processes (Bulun *et al.*, 2021; Guo, 2023; Kobayashi, 2023). These events may disrupt the endo-myometrial junction, favouring intra-myometrial invagination of basal endometrium fragments and the development of adenomyosis (Jarrell, 2018; Bulun *et al.*, 2021). Platelet aggregation and macrophages recruitment would contribute to inflammation, local oestrogen synthesis, tissue remodelling, and ultimately fibrogenesis (Guo, 2028, 2020, 2023).

In summary, repetitious ovulatory menses may favour the displacement of endometrial fragments in the pelvis via transtubal retrograde flow and into the myometrium via the opening of a ‘denuded intrauterine terrain’. The excess of free iron resulting from repetitive bleeding episodes may damage the mesothelial lining, which would facilitate pelvic implantation of endometrial glands, and may induce resistance to ferroptosis, which would trigger lesion progression and eventually fibrosis (Li *et al.*, 2023; Wyatt *et al.*, 2023; Wu *et al.*, 2023). In addition, relentless ovulation increases oestrogen exposure, with the consequent stimulation to proliferation and infiltration of ectopic endometrium.

## Criticisms of the repetitive ovulatory menstruation hypothesis

There is a large body of evidence to support the hypothesis that ROM contributes to the development of endometriosis and adenomyosis, but we did not conduct a formal systematic review of the literature, so our search may have missed important studies. Some of the publications considered are old, and the quality of the included reports was not assessed. Moreover, we disclose our intellectual conflict of interest related to the belief that retrograde menstruation is the necessary, although not sufficient, condition for the development of most cases of endometriosis. Finally, our hypothesis is based on the change in reproductive patterns observed in Western countries. The situation may be different in countries with different ethnic groups (Somigliana *et al.*, 2012; Ohayi *et al.*, 2022). In addition to the these methodological issues, there are three main criticisms that can be made of our ROM hypothesis.

(i) First, there is no evidence of an increase in the incidence of endometriosis and adenomyosis following the demographic transition. Neither disease could be diagnosed in the pre-industrial era, but an increase in the incidence of endometriosis as a result of an increase in the number of ovulatory menstruations remains unproven even in recent decades (Christ *et al.*, 2021). In fact, in a systematic review of data published between 1989 and 2019, Ghiasi *et al.* (2020) observed no time trend in the incidence and prevalence of endometriosis in the general population. Methodological limitations prevented the authors from drawing conclusions about a possible change in the severity of incident cases over the past 30 years.

However, several biases preclude an accurate definition of the true frequency of endometriosis, including diagnostic bias leading to underestimation in population studies, and health inequalities influencing access to care and assessment by physicians with sufficient knowledge of the condition. Geographical, economic, and social factors also affect the possibility of being evaluated by an experienced imaging specialist (Ghiasi *et al.*, 2020). An excellent overview of the dynamics involved in the pathways to endometriosis diagnosis has been proposed by Shafrir *et al.* (2018).

Moreover, it seems reasonable to assume that most of the changes in reproductive patterns in Western countries in the post-industrial era have already occurred half a century ago (Short, 1976, 2002; Eaton *et al.*, 1994; Strassmann, 1996, 1997). In fact, compared with the changes that had taken place over the previous two centuries, there have been only minor variations over the last 50 years, as the fertility rate has remained essentially stable. For example, the total fertility rate in the USA was 7 in 1800, 3.9 in 1900, fell below the replacement level in the early 1970s, and has remained at around 2 ever since (United Nations Department of Economic and Social Affairs, Population Division, 2019). Thus, it cannot be excluded that the effect of an increase in the number of ovulatory menstruations was already almost fully established when awareness of endometriosis and adenomyosis increased and diagnostic motivation and competence improved in the 1980s. If this is the case, contemporary epidemiological studies would not be expected to reveal major changes in the incidence of these diseases over time, but this would not per se discredit the possibility of an earlier and therefore undetected effect of an increased number of ovulatory menstruations on the risk of endometriosis and adenomyosis.

(ii) Second, in addition to repetitious ovulatory cycles, several other factors appear to play a role in the pathogenesis of endometriosis and adenomyosis, including genetic predisposition, epigenetic profile, constitutional variables, immunological and hormonal factors, and individual lifestyle (Shafrir *et al.*, 2018; Zondervan *et al.*, 2018, 2020; Bulun *et al.*, 2019, 2021, 2023; Guo, 2020, 2023; WaNg *et al.*, 2020; Taylor *et al.*, 2021; Horne and Missmer, 2022; Vallée *et al.*, 2023). Thus, even if there is an actual progressive increase in frequency, this could be explained by augmented exposure to toxic environmental chemicals and endocrine disruptors (Sirohi *et al.*, 2021; Matta *et al.*, 2021; Vallée *et al.*, 2023; Marroquin *et al.*, 2023), high trans-unsaturated fat intake (Missmer *et al.*, 2010), altered microbiome (Koninckx *et al.*, 2019; Leonardi *et al*, 2020; Salliss et al., 2021; Muraoka *et al.*, 2023; Yang *et al.*, 2023), or other features of modern life that may influence the risk of these diseases (Missmer and Cramer, 2003; Viganò *et al.*, 2004; Ottolina *et al.*, 2020; Sasamoto *et al.*, 2020;  Shafrir *et al.*, 2018; Koninckx *et al.*, 2021a). More in general, since endometriosis and adenomyosis appear as multistep phenomena in which, after an establishment phase, a proliferation and invasion phase occurs, accompanied or followed by an inflammatory reaction phase, each phase may be subject to the influence of different contributing factors (Parazzini *et al.*, 2017).

Furthermore, the real question here may not necessarily be: ‘Are there other factors that could explain an [unobserved] increase in the incidence of endometriosis and adenomyosis in WEIRD societies?’ But rather, ‘Are there confounding factors of the demographic transition that could explain the link between ROM and endometriosis and adenomyosis, thus negating the causal relationship between ROM and endometriosis and adenomyosis?’ As a theoretical example, a contemporary change in diet could have led on the one hand to an earlier menarche (Gamble, 2017) and, on the other hand, to an increased risk of endometriosis due to a higher intake of selected nutrients such as red meat, trans fatty acids, and saturated fatty acids (Missmer *et al.*, 2010; Arab *et al.*, 2022). Thus, a rise in the number of ROMs is identified, but the pathogenic factor causing the increase in the onset of endometriosis is actually the change in diet over time. A different epidemiological context is represented by the increasing use over the last century (Barton, 1942; Bullough, 1985; Liberty *et al.*, 2023) of thousands of menstrual pads and tampons per person, as these disposable products have been shown to contain and release several endocrine disruptors, such as phthalates, phenols, and parabens, which may promote the development of endometriosis and adenomyosis (Marroquin *et al.*, 2023). In this case, the causative factor would not be the recent increase in ROM per se, but the resulting contemporary increased exposure of the average menstruator to chemicals that can be absorbed by the vulvar and vaginal mucosa without undergoing first-pass metabolism (Marroquin *et al.*, 2023). In addition, heavy menstrual flow has been consistently associated with the risk of endometriosis (Cramer *et al.*, 1986; Parazzini *et al.*, 1995; Matalliotakis *et al.*, 2008). At the same time, women with menorrhagia tend to use higher absorbency products and more protective disposables per cycle, thus potentially increasing their exposure to endocrine-disrupting chemicals (Marroquin *et al.*, 2023).

(iii) Third, probably the most important weakness of our theory is that exposure to retrograde menstruation appears to be the norm for most women (Halme *et al.*, 1984; Liu and Hitchcock, 1986; Kruitwagen, 1993; Tang *et al.*, 2022), but the prevalence of endometriosis and adenomyosis is relatively low (Missmer and Cramer, 2003; Parazzini *et al.*, 2017, 2020; Ghiasi *et al.*, 2020; Sarria-Santamera *et al.*, 2020; Christ *et al.*, 2021). Thus, it seems logical to accept that protective mechanisms against the potential effects of ROM are likely to be at play, otherwise almost all menstruators would sooner or later develop endometriosis during the reproductive period. Presumably, the variable individual level of antioxidant potential of pelvic macrophages may determine whether a given amount of refluxed erythrocytes and endometrial glands can overwhelm the scavenging capacity, thus allowing damage to the mesothelial lining and implantation of the refluxed endometrium (Van Langendonckt *et al.*, 2002a,b; Lousse *et al.*, 2009, 2012; Donnez *et al.*, 2016; Ng *et al.*, 2020; Ansariniya *et al.*, 2022; Wyatt *et al.*, 2023). Furthermore, different patterns of crosstalk between pelvic NK lymphocytes and the local microenvironment may determine whether refluxed endometrial epithelial cells harbouring somatic cancer driver mutations (Praetorius *et al*, 2022) are systematically eliminated or undergo immune escape (Liang *et al.*, 2019; Fukui *et al.*, 2021).

However, some more clarity on this point might also be useful to put the ROM hypothesis in the right context. Apart from the seminal articles by Sampson (1927, 1940), which still form the solid foundation of the implantation theory (Yovich *et al.*, 2020), and some anecdotal reports published before the 1980s (for review, see Kruitwagen, 1993; D’Hooghe and Debrock, 2002), the first study to document the systematic and not occasional occurrence of retrograde menstruation, was published by Blumenkrantz *et al.* (1981). The authors reviewed data from 11 menstruators between the ages of 15 and 44 who were undergoing periodic peritoneal dialysis for end-stage renal failure. In nine of them, blood was regularly observed in the Silastic peritoneal dialysis catheter just before menses and on the first day of menstruation. Bloody peritoneal fluid was never observed at any other time during the menstrual cycle. Six patients underwent laparotomy for various reasons, but no endometriosis was found. Numerous attempts to identify endometrial cells or glands in the peritoneal effluent of three patients were unsuccessful.

Halme *et al.* (1984) aspirated peritoneal fluid from 331 patients during laparoscopy and classified it as straw-coloured or pink or bloody. Of the 65 patients who underwent surgery on perimenstrual days (1–6 and 27–30 of the cycle), 49 (75%) had pink or bloody fluid (38/42 without endometriosis and with open tubes; 2/11 without endometriosis and with closed tubes; and 9/10 with endometriosis). Of relevance, eight of the nine patients who underwent laparoscopy in the perimenstrual phase while on cyclic oral contraceptives had bloody peritoneal fluid. However, peritoneal fluid samples were examined for the presence of blood, but not for endometrial cells or glands.

Inspired by these landmark studies, which showed that transtubal blood reflux during menses was very common, several investigators began to focus on the identification of endometrial cells or tissue structures in the peritoneal fluid in addition to the presence of blood (Kruitwagen, 1993; D’Hooghe and Debrock, 2002).

Reti *et al.* (1983) performed laparoscopy during menstruation in 15 symptomatic (mean age 26.2 years) and 31 asymptomatic (35.4 years) individuals. Bloodstained peritoneal fluid was aspirated in 10 (67%) subjects of the former group and in 23 (74%) of the latter. Small dense clusters of cells resembling endometrial glandular and stromal material were identified cytologically in five (33%) and three (10%) cases, respectively. Endometriosis was detected in only one symptomatic patient.

In subsequent studies, different techniques were used, including not only cytology, but also cell block analysis, immunocytochemistry, and cell culture (Kruitwagen, 1993), and high variability was observed in the occurrence of peritoneal fluid samples positive for endometrial cells or tissue fragments, with percentages varying from 19% to 75% when endometriosis was present and from 10% to 92% when it was not (Kruitwagen, 1993).

This inconsistency in the observed frequencies, together with the limited sample size of most studies, the heterogeneity of the methodological approaches used, the collection of samples at different phases of the cycle, and the choice of highly selected participants who may not be representative of the general population (e.g. infertile patients with patent and blocked fallopian tubes, or parous people undergoing tubal sterilization), may limit the validity of the available data. In fact, the frequency of the perimenstrual presence of endometrial cells or glands, and not merely ‘blood’, in the peritoneal fluid of menstruators in the general populations, and not just in selected patients undergoing surgery for different indications, is currently undefined (Bokor *et al.*, 2009; Dorien *et al.*, 2017).

Thus, the idea that true retrograde menstruation is the norm during the reproductive years has not been conclusively proven and cannot be taken for granted (Bartosik *et al.*, 1986). It would be extremely helpful to know how many menstruators have transtubal reflux containing endometrial cells or glands, whether this occurs occasionally or every month, how many days it lasts, what the absolute amount of refluxed endometrial cells is, whether the cytological characteristics remain stable over time or vary, and what, if any, differences there are between individuals who develop endometriosis and those who do not, both in the erythrocyte component (potentially responsible for the oxidative stress) and in the mucosal component. The mere observation of perimenstrual transtubal blood reflux cannot be considered synonymous with demonstrating the validity of the metastatic theory (Kruitwagen, 1993; van der Linden *et al.*, 1995) but, with few exceptions (e.g. Bulletti *et al.*, 2002; Bokor *et al.*, 2009; Dorien *et al.*, 2017; Masuda *et al.*, 2021; Tang *et al.*, 2022), research into this pathogenic aspect seems to have rather waned in the last two decades.

Overall, it seems premature to exclude the possibility that elementary mechanistic factors (e.g. calibre of the cervical canal, internal cervical os stiffness, calibre of the tubal ostia, diameter and course of the intramural tubal portion, function of the utero-tubal junction, amount of menstrual flow, frequency and strength of myometrial contractions with loss of fundocervical polarity) (Bartosik *et al.*, 1986; Cramer *et al.*, 1986; Barbieri *et al.*, 1992; Vercellini *et al.*, 1997; Barbieri, 1998; Leyendecker *et al.*, 2004; Bulletti *et al.*, 2002; Missmer *et al.*, 2004; Rozewicki *et al.*, 2005; Munro, 2023; XholLi *et al.*, 2023) together with non-modifiable genetic determinants (Liang *et al.*, 2023) govern the amount of refluxed blood and endometrium per cycle in the general population of menstruators and thus contribute substantially to the chance of developing endometriosis in specific subgroups (D’Hooghe and Debrock, 2002; Yovich *et al.*, 2020). Indeed, endometriosis is significantly more common in patients with obstructive Müllerian anomalies than in those with non-obstructive anomalies (Pinsonneault and Goldstein, 1985; Sanfilippo *et al.*, 1986; Olive and Henderson, 1987). In addressing the question ‘*Why do not all women develop endometriosis?*”, ’ D’Hooghe and Debrock (2002) stated, after reviewing published epidemiological and experimental data, that ‘*it can be hypothesised that the quantity of retrogradely flushed endometrial cells may be important in the development of endometriosis and in the spontaneous evolution of the disease*’.

Ultimately, it remains uncertain whether the association between ROM and endometriosis-adenomyosis can be interpreted as causal. In an attempt to disentangle this issue, the Bradford Hill criteria (1965) could be used as a framework. However, these criteria, defined as ‘viewpoints’ by the author himself (Hill, 1965), are not intended to be a checklist to support causation, and meeting all the criteria is neither necessary nor sufficient to establish it. Furthermore, alternative hypotheses to explain the association presented here cannot be ruled out (Table 2). In fact, especially in multifactorial diseases, causation can rarely be established irrefutably, and a range from ‘highly unlikely’ to ‘highly likely’ is more often contemplated (Nowinski *et al.*, 2022).

**Table 2.** Applying the Bradford Hill (1965) criteria for causation to reiterative ovulatory menstruation and endometriosis-adenomyosis.

Criterion	Strength of evidence*	Argument for fulfilment	Supporting citations
Strength	++	Consistent associations have been observed between age at menarche, regular cycles, and heavy menstrual flow, i.e. indicators of pelvic exposure to refluxed endometrium, and endometriosis	Cramer *et al.* (1986), Darrow *et al.* (1993), Parazzini *et al.* (1995, 2017), Vercellini *et al.* (1997), Missmer and Cramer (2003), Missmer *et al.* (2004), Viganò *et al.* (2004), Treloar *et al.* (2010), Nnoaham *et al.* (2012), Hoppenbrouwers *et al*. (2016), Shafrir *et al.* (2018), and Lu *et al.* (2023).
Consistency	++	The above associations have been observed by independent groups in different countries	Missmer and Cramer (2003), Missmer *et al.* (2004), Viganò *et al.* (2004), Nnoaham *et al.* (2012), Hoppenbrouwers *et al*. (2016), Parazzini *et al.* (2017), Shafrir *et al.* (2018), and Lu *et al.* (2023).
Specificity	++	Although exceptions have been described, in the vast majority of cases adenomyosis and endometriosis develop and progress in menstruators. The distribution of endometriotic lesions is consistent with the dissemination of refluxed endometrial glands according to anatomical and physiological determinants	Vercellini *et al.* (1998, 2007, 2014), Missmer and Cramer (2003), Viganò *et al.* (2004), Parazzini *et al.* (2017, 2020), and Shafrir *et al.* (2018).
Temporality	+++	Excluding anecdotal cases, adenomyosis and endometriosis are diagnosed after a variable number of years of repetitive ovulatory menstruations	Missmer and Cramer (2003), Missmer *et al.* (2004), Parazzini *et al.* (2017), Shafrir *et al.* (2018), Zondervan *et al*. (2018, 2020), Bulun *et al.* (2019, 2021, 2023), Guo (2020, 2023), Wang *et al.* (2020), Koninckx *et al.* (2021b), Taylor *et al.* (2021), Horne and Missmer (2022), and Kobayashi (2023a).
Biological gradient	++	A dose–response relationship has been observed between the number of ovulatory menses and the amount of menstrual flow and the risk of endometriosis	Darrow *et al.* (1993), Vercellini *et al.* (1997), Missmer and Cramer (2003), Missmer *et al.* (2004), Viganò *et al.* (2004), Matalliotakis *et al.* (2008), and Parazzini *et al.* (2017).
Plausibility	+++	An large amount of data supports the mechanistic role of reiterative ovulatory menstruation in the pathogenesis of both adenomyosis and endometriosis	Zondervan *et al.* (2018, 2020), Bulun *et al.* (2019, 2021, 2023), Guo (2020, 2023), Wang *et al.* (2020), Koninckx *et al.* (2021b), Taylor *et al*. (2021), Horne and Missmer (2022), and Kobayashi (2023a).
Coherence	++	Epidemiological observations are consistent with several laboratory studies	Greaves *et al.* (2014), Shen *et al.* (2016), Zhang *et al.* (2017), Kusama *et al.* (2021), and Cordeiro *et al.*, (2022).
Experiment	+	Experimental evidence exists for the validity of pelvic iron overload and secondary oxidative stress as determinants of chronic inflammation and fibrosis. No randomized, controlled trials have been conducted on the effect of early menstrual suppression and risk of endometriosis and adenomyosis	Van Langendonckt *et al.* (2002a,b), Lousse *et al.* (2009, 2012), Donnez *et al.* (2016), Ng *et al.* (2020), Ansariniya *et al.* (2022), Kobayashi (2023b), and Wyatt *et al.* (2023).
Analogy	++	The same pathogenic mechanism (i.e. retrograde menstruation) is supported by epidemiological and laboratory findings in different benign (endometriosis and adenomyosis) and malignant (serous, endometrioid and clear cell ovarian cancer) diseases. In addition, exposure for decades to high oestrogen levels due to reiterative ovulations facilitates the onset of both benign and malignant hormone-sensitive diseases such as endometriosis and adenomyosis as well as breast and endometrial cancer.	Macmahon *et al.* (1982), Vihko and Apter (1984), Viganò *et al.* (2004), Vercellini *et al.* (2006, 2011), Seidman (2013), Lattuada *et al.* (2015), Huang *et al.* (2016), Uberti *et al.* (2016), Farland *et al.* (2017b), Rockfield *et al.* (2019), Chhabra *et al.* (2021), and Bieuville *et al.* (2023).

* Strength of evidence: + weak; ++ moderate; +++ strong.

With due regard to the above caveats, based on the available evidence, the association between ROM and endometriosis-adenomyosis appears to be moderately skewed towards ‘highly likely’ on the continuum between the two extremes of the causation scale (Table 2). Moreover, even if additional pathogenic causes are eventually identified, they would not necessarily diminish the importance of the relationship between ROM and endometriosis-adenomyosis.

Against this background, the next key question is whether medical interventions with an appropriate balance of potential benefits, potential harms, and costs could be implemented in practice to try to limit the risk of progression of early endometriotic and adenomyotic lesions to more extensive forms, with the consequent potential detrimental effects on health-related quality of life and fertility.

## Prospectus: preventive interventional endocrinology?

The available epidemiological evidence on the possible effect of ROM on the frequency of endometriosis may appear puzzling, as on the one hand it consistently shows convincing associations between menstrual patterns suggesting increased retrograde pelvic contamination (e.g. early menarche, short and regular cycles, abundant menses) and the disease (Missmer and Cramer, 2003; Missmer *et al.*, 2004; Viganò *et al.*, 2004; Parazzini *et al.*, 2017; Shafrir *et al.*, 2018), but on the other hand it does not indicate an increase in the incidence and prevalence of endometriosis over time in regular menstruators (Ghiasi *et al.*, 2020; Parazzini *et al.*, 2020; Christ *et al.*, 2021; Sarria-Santamera *et al.*, 2020).

Indeed, research into retrograde menstruation should be resumed, as it seems to have been somewhat disregarded, despite the possible fundamental pathogenic insights that may be derived from it (Wyatt *et al.*, 2023). In fact, uncertainties about the potential impact of the substantial increase in the number of ovulatory menstruations in the post-industrial era on the risk of endometriosis appear to be partly determined by our limited knowledge of what actually happens in the pelvis of individuals in the general population during the perimenstrual days.

However, despite the criticisms of our view described above, it is undeniable that menstruators from Palaeolithic to pre-industrial times spent the first decade after menarche in a relatively stable hormonal environment, characterized by infrequent ovulatory menstruation, prolonged progesterone exposure and long periods of hypoestrogenism, whereas menstruators from Western, industrialized, high-income nations now experience recurrent gonadal hormone fluctuations, characterized by repetitious ovulatory menstruation, prolonged oestrogen exposure, and the absence of hypoestrogenic phases. Indeed, Renfree (2012) suggests that reiterative menstruation is an iatrogenic disorder of modern societies, potentially causing ‘*diseases of nulliparity*’, and several investigators support the notion of endometriosis and adenomyosis as disorders initiated by ROM in susceptible individuals, promoted by a mitogenic and pro-inflammatory hyper-oestrogenic local and systemic milieu, and alleviated by amenorrhoea and long-term, stable, low-oestrogen, and high-progesterone exposure (Kruitwagen, 1993; D’Hooghe and Debrock, 2002; Lousse *et al.*, 2009; Donnez *et al.*, 2016; Bulun *et al.*, 2019, 2021, 2023; Guo, 2020, 2023; Maruyama *et al.*, 2020; Koninckx *et al.*, 2021c; Bulun, 2022;  Yovich *et al.*, 2020; Kobayashi, 2023; Kobayashi *et al.*, 2023; Wyatt *et al.*, 2023).

Given these premises, should we ultimately accept or reject the null hypothesis that ROM has no effect on disease development and progression? From an evolutionary point of view, should not anovulation and amenorrhoea still be considered the physiological state at least during the early reproductive years? If ROM, especially during adolescence, defined here as the period between 12 and 20 years of age (Martire *et al.*, 2020; Millischer *et al.*, 2023), has an impact on the likelihood of the development and progression of endometriosis and adenomyosis, then there could be an opportunity to use hormonal interventions in secondary prevention selectively in individuals with disabling pelvic pain to reduce the burden of exposure to one of the well-established risk factors for these two conditions (Fathalla, 2019; Bulun *et al.*, 2019, 2021; Munro, 2023).

Notwithstanding foreseeable problems with diagnostic modalities, recruitment, and long-term treatment adherence, randomized trials could be designed to provide mechanistic insight into whether inhibiting ovulation and menstruation can protect adolescents with severe dysmenorrhoea and heavy monthly bleeding from progression of early-onset endometriosis and adenomyosis. However, this would take many years and, in the meantime, the ethical question may emerge as to whether, in the absence of definitive epidemiological evidence of causation and results from formal RCTs, we are justified in not suppressing a hypothetical source of lesions and an undeniable cause of symptoms.

In the words of Nowinski *et al.* (2022) ‘*any actions are risky, including decisions to do nothing. When thinking of causation as a continuum from highly unlikely to highly likely, we must consider the expected net harm of doing something vs. doing nothing. If we do something, and we learn later that the hazard was not really a hazard, there may be harm done. If we do nothing, and the hazard was in fact a hazard, there will be harm done of a very different sort*’.

The issue may have important clinical implications given that (i) most adolescents with severe pelvic pain symptoms have endometriosis (Janssen *et al.*, 2013); (ii) most lesions visualised at laparoscopy are limited superficial peritoneal implants (Hirsch *et al.*, 2020); (iii) a not negligeable proportion of these superficial lesions will progress to more severe and fibrotic forms (Koninckx *et al.*, 2021b); (iv) the consequences of disease progression are potentially severe.

Accordingly, in the second part of this opinion piece, a proposal for possible individualized secondary prevention of early-onset endometriosis and adenomyosis is presented based on the evolutionary considerations expressed above.

## Data Availability

The data included in this article were extracted as published in the available original articles. No new data were generated or analysed to support this article.
